# Medicinal plants used in traditional treatment of malaria in Ethiopia: a review of ethnomedicine, anti-malarial and toxicity studies

**DOI:** 10.1186/s12936-022-04264-w

**Published:** 2022-09-10

**Authors:** Gashaw Nigussie, Minychel Wale

**Affiliations:** 1 Department of Biotechnology and Bioinformatics, Armauer Hansen Research Institute, Post Box 1005, Addis Ababa, Ethiopia; 2 Department of Pharmacy, All Africa Leprosy, Tuberculosis and Rehabilitation Training Centre, Addis Ababa, Ethiopia

**Keywords:** Antimalarial, Ethnomedicine, Medicinal plants, Ethiopia

## Abstract

**Background:**

Malaria is extremely common in Ethiopia, and it is one of the country's most serious public health and economic problems. Traditional medicines have long been utilized in Ethiopia by people of various ethnic groups. As a result, the goal of this study is to record the use of Ethiopian medicinal herbs that have been used to treat malaria. Also, a critical review of the literature on the therapeutic properties of these and other Ethiopian medicinal plants that have been tested against *Plasmodium* spp*.* parasites was conducted with the goal of highlighting neglected studies and fostering further research in this area.

**Methods:**

A comprehensive literature search was performed in Scopus, Web of Science Core Collection, PubMed, Science Direct, Google Scholar, and Scientific Electronic Library Online (SciELO) from August 2021 to October 2021. The study databases included original articles published in peer reviewed journals covering anti-malarial plants, dated until October 2021.

**Results:**

The review looked at 51 plant species (28 families) that have been used to treat malaria in Ethiopia. The most often used ethnobotanical plant species for the treatment of malaria were *Allium sativum*, *Croton macrostachyus*, *Carica papaya*, and *Lepidium sativum*. Leaves were used more frequently as a therapeutic preparation than other parts. Plant extracts were found to have very good, good, and moderate anti-malarial activity in mice with rodent *Plasmodium* species. The most active species were *Ajuga remota* and *Capsicum frufescens*, which suppressed parasitaemia by 77.34% and 72.65%, respectively, at an oral dose of 100 mg/kg and an LD_50_ of above 2000 mg/kg. The compound Aloinoside reported from *Aloe macrocarpa* leave latex was the most potent; it suppressed parasitaemia by 100% at 400 mg/kg oral dose of *Plasmodium berghei* infected mice, and its LD_50_ was above 2000 mg/kg. Toxicity was shown to be safe in 84% of the plant extracts.

**Conclusion:**

In Ethiopia, medicinal plants have a significant part in reducing the severity of malaria due to their widespread use. As a result, more studies are needed to identify and develop effective novel drugs that could be employed in broader malaria eradication efforts.

## Background

Malaria is a disease caused by *Plasmodium* parasites and it is one of the primary causes of death and morbidity in many undeveloped countries [1]. Malaria affects an estimated 3.3 billion people globally [2], and it is a major public health issue in tropical and subtropical areas [3]. According to the World Health Organization (WHO), 229 million new cases of malaria were reported worldwide in 2019, and an estimated 409,000 people died from malaria in that period. The majority of malaria cases and resulting mortality occurred in the WHO African area (94%) [4]. Malaria causes major complications in infected people, such as severe anaemia, cerebral malaria, acute renal failure, and hypoglycaemia [5]. Five *Plasmodium* species are responsible for the disease [6] and four of these species occur in Ethiopia—*Plasmodium falciparum*, *Plasmodium vivax*, *Plasmodium ovale*, and *Plasmodium malariae* [7].

*Plasmodium falciparum* is the most severe *Plasmodium* species in terms of morbidity and mortality, followed by *Plasmodium vivax* with proportions of 60% and 40%, respectively [8]. Malaria is one of Ethiopia's most serious public health and economic issues. The prevalence of malaria in children and pregnant women are 0.6% and 16.3%, respectively [9, 10]. In Ethiopia, the transmission patterns and intensity vary greatly due to the large diversity in altitude, rainfall, and population movement, with areas below 2000 m being potentially malarious. Those areas are home to approximately 68% (52 million) of the Ethiopian population and cover almost 75% of the country's landmass, resulting in around 10 million clinical cases each year according to Ethiopian National Malaria Indicator survey of 2007 [11]. Ethiopia is one of the countries that have adopted the revised malaria control strategies. The most crucial in malaria prevention and control strategies are indoor residual spraying and long-lasting insecticidal nets. In Ethiopia, quick diagnostic tests are being introduced at the community level, as well as the adoption of artemisinin-based combination therapy (ACT). However, there have already been instances of increased treatment failure and probable resistance to certain combinations [12–14]. As a result, new medications as prospective substitutes for artemisinin-based combinations are urgently needed. Pharmaceutical firms, on the other hand, consider a large investment in the development of new (semi)synthetic anti-malarial medications to be a dangerous venture, because the populations of developing nations cannot afford to pay a high price for these drugs. There is a need to develop new cost effective anti-malarial drugs to assist in controlling malaria and reducing its impact in these areas until eradication programmes become realistic.

One approach to the development of novel anti-malarial drugs is to reinvestigate traditional medicines. In this context, Ethiopia possesses a diverse range of medicinal plants linked to a variety of traditional medical practices that vary by ethnic group [15]. Despite this, there is a paucity of well-documented ethnobotanical and ethnopharmacological literature on Ethiopian anti-malarial herbs. The review looked at the various ethnomedicinal studies that have been conducted, as well as the scientific validation of antiplasmodial activity, anti-malarial activity, toxicity, and phytochemistry of these plants utilized in Ethiopian traditional medicine. This review may open the way for additional supplementary research as well as the development of a number of readily available and affordable anti-malarial phytomedicines, in keeping with the goals of the WHO's "Traditional Medicine Strategy" [16].

## Methods

A comprehensive literature search was performed in Scopus, Web of Science Core Collection, PubMed, Science Direct, Google Scholar, and Scientific Electronic Library Online (SciELO) from August 2021 to October 2021. The search was performed independently in all databases. The study databases included original articles published in peer reviewed journals covering anti-malarial plants, dated until October 2021.

Articles offering information on malaria or medicinal plants in Ethiopia were given utmost priority throughout all publishing years. As a result, references found in the returned results were evaluated for inclusion in this study, and further searches were conducted using more general search terms such as "Ethiopian," "medicinal plant," "traditional medicine," "ethnomedicine," "parasite," "malaria," "anti-malarial," and "antiplasmodial". The study was non-biased, with no preference for endemic species or taxonomic preference. The initial ethnobotanical literature search did not include scientific evidence to support traditional use, but it was added in subsequent searches to see if the traditional use had been validated. The search was restricted to studies that were written in English. Relevant articles were identified and the data extracted by the reviewers: plant species, plant family, parts of the plant used, methods of preparation, type of study (whether in *vitro* or in *vivo*), the extraction solvent used, IC_50_ or ED_50_ values, parasite suppression rate, isolated compounds, strain of *Plasmodium* tested and toxicity.

### Categorization of anti-malarial and antiplasmodial activities

For in *vitro* investigations, antiplasmodial activity of extracts was rated very good if the IC_50_ was less than 5 μg/ml, good if the IC_50_ was greater than 5 μg/ml and less than 10 μg/ml, and moderate if the IC_50_ was 10 μg/ml ≤ IC_50_ < 20 μg/ml [17]. For in *vivo* investigations, an extract's anti-malarial activity is deemed very good if it suppresses malaria by ≥ 50% at 100 mg/kg body weight/day, good if it suppresses malaria by ≥ 50% at 250 mg/kg body weight/day, and moderate if it suppresses malaria by ≥ 50% at 500 mg/kg body weight/day [17].

## Results and discussion

### Ethiopian medicinal plants used traditionally to treat malaria

Ethiopia has a diverse flora, and some local people employ several of the plant species for medical purposes [18]. The widespread use of traditional medicines in Ethiopia can be linked to its cultural acceptability, efficacy against specific ailments, physical accessibility, and economic affordability when compared to modern medicine [19]. Traditional remedies are the most important and, in some cases, the only source of treatments for approximately 80% of Ethiopians, and 95% of the preparations are made from plants [19]. In different locations of Ethiopia, 51 plant species from 28 families were reported as being engaged in the treatment of malaria (Table 1). The following families account for 64% of the anti-malarial plant species documented across the country: *Fabaceae* has nine species, *Asteraceae* has five, *Euphorbiaceae* has four, *Aloaceae* has three, *Solanaceae* has three, *Alliaceae* has two, *Urticaceae* has two, and *Meliaceae* has two species. *Allium sativum*, *Croton macrostachyus*, *Carica papaya*, and *Lepidium sativum* were the most commonly employed ethnobotanical plant species for the treatment of malaria (Table 1).

**Table 1:** Ethiopian medicinal plants used traditionally to treat malaria

Family^a^	Plant species	Local name	Parts used	Methods of preparation	References
*Alliaceae* (2)	*Allium sativum*	Nech shinkurt (A)	Steams	Peeling the cover then eat with nutrient	[25]
			Bulbs	The bulb, which is free of external scales, is crushed and blended with honey before being consumed on an empty stomach	[26]
			Bulbs	*Allium sativum* bulb and *Ginger officinale* rhizome are pounded and eaten with honey	[27]
			Fruits	Fresh or dry fruits is chewed orally	[28]
			Bulbs	Before eating breakfast, take the bulb with Ethiopian traditional food 'injera' and *Capsicum annuum* for 5 days	[18]
			Fruits	Crush the fruit and boil it, then drink it with much amount of milk for 1 day	[29]
			Bulbs	Crush it and consume it alone or mixed with *Lepidium sativum* seeds	[30]
	*Allium cepa*	Keye shinkurt (A)	Bulbs	Chew the bulbs and swallow it	[31]
*Aloaceae* (3)	*Aloe gilbertii*	Kurunda (Had)	Leaves sap	Squeezed fresh leaves soup and taking the soup orally	[32]
	*Aloe sp.*	Yeset qest (A)	Leaves	Fresh leaves were squeezed and diluted with water and drunk it. Syrup made from the plant's dried leaves, as well as those of *Asparagus africanus* and *Senna italica*, is also drunk	[33]
	*Aloe megalacantha*	Ere (T)	Leaves	Crush the leaves to get the juice, then filter and drink the filtrate	[30]
*Asteraceae* (5)	*Artemisia abyssinica*	Aritimiza (Had)	Leaves	Fresh leaves were crushed and pounded with water, filtered and drunk until they were recovered	[34]
	*Vernonia amygdalina*	Grawa (A)	Leaves and barks	For days, morning and evening, leaves and bark mixed with honey are consumed	[35]
			Leaves	Crushed leaves of *Vernonia amygdalina* concocted with leaves of *Ruta chalepensis*. One cup is served as a drink for 3–5 days with cold water in the morning	[22]
	*Artemisia afra*	Chugughee (A)	Leaves	Powdered fresh/dry leaves mixed with butter is taken with coffee orally before breakfast for three days	[28]
			Leaves	Fresh leaves crushed and pounded with water and then filtered and drunk in one tea cup	[36]
	*Calpurnia aurea*	Digita (A)	Leaves	Maceration, taken orally once daily for seven days	[37]
	*Echnops kebericho*	Kebericho (A)	Roots	Maceration; take orally once daily for seven days	[37]
*Boraginaceae* (1)	*Cordia africana*	Wanza (A)	Roots and Barks	Decoction of roots and inner bark with ginger is consumed	[35]
*Brassicaceae* (1)	*Lepidium sativum*	Feto (A)	Fruits	Dried fruit is ground into powder, mixed with castor oil, and administered orally	[35]
			Seeds	Pounded seeds mixed with *Allium sativum* bulbs and honey is taken orally for five days before breakfast After each dose, one glass of melted butter is recommended for immediate recovery	[28]
			Seeds	The seed is powdered and made as Porridge with other grains	[38]
*Capparidaceae* (1)	*Maerua oblongifolia*	Ja”a (O)	Leaves	Pounded leaves boiled with goat milk and drunk. It is also taken in mixture with the leaves of *Withania somnifera*	[33]
*Caricaceae* (1)	*Carica papaya*	Papaya (A)	Leaves	Squeezed the fresh leaves juice and drunk	[39]
			Leaves	The fresh leaves crush and drink with milk or without milk	[36]
			Leaves	When the leaves become yellow, that means getting to dry, powdered and boiled in water and a cup of tea will be taken for 5 days	[18]
			Leaves	Leaves are pounded and then boiled; the decoction is taken while cold	[38]
*Caryophyllaceae* (1)	*Silene macrosolen*	Saerosaero (T)	Roots	Crush and place it on fire for fumigation	[30]
*Combretaceae* (2)	*Combretum molle*	Agalo (A)	Leaves and Barks	Leaves and barks powder are mixed with tea or coffee and drunk	[35]
	*Terminalia brownie*	Sebaea (T)	Barks	The fresh barks of *Terminalia brownie* pounding, homogenize with water and drink a bottle cup of the decant in the morning in empty stomach for 4 days	[40]
*Convolvulaceae* (1)	*Ipomoea kituiensis*	Laalata (O)	Leaves	Juice of fresh leaves is drunk with coffee	[35]
*Cucurbitaceae* (1)	*Lagenaria siceraria*	Buqqe hadhaa (O)	Fruits	Ripe fruit of *Lagenaria siceraria* is bored rinsed with cold water, one glass is used as a drink early in the morning	[22]
*Euphorbiaceae* (4)	*Croton macrostachyus*	Bisana (A)	Leaves	Boil fresh leaf in water, filter, and drink with milk or tea	[36]
			Leaves	Macerate with water; take two doses orally for one day	[37]
			Leaves	Crushed/pounded fresh/dry leaves boiled with water is concocted with *Allium sativum* (bulb) roasted with butter and left over night outside home is taken orally at the morning	[28]
			Leaves	Powdered leafy-stem of *C. macrostachyus* is mixed with H_2_O and butter and drank the filtrate part	[41]
			Leaves	Crushing leaves and drinks with either *Guizotia abyssinica* or milk	[29]
	*Euphorbia abyssinica*	Kulkual (A)	Latexs	Fresh latex of *Euphorbia abyssinica* eat bake with *Eragrostis* tef dough	[26]
	*Jatropha curcas*	Habet-muluk (So)	Leaves	The outer cover of the seed removed and the inside part swallowed with camel milk or chewed	[33]
	*Euphorbia abyssinica*	Kulkual (A)	Roots	Crushing the root and drink with milk	[29]
*Fabaceae* (9)	*Sesamum indcum*	Eshkulubia (Ku)	Roots	Pounding the fresh root, mixed with boiled milk and drink a half cup of it in the morning and afternoon	[40]
	*Tephrosia gracilipes*	Atotoka (Ku)	Roots	Crushing the dried root, homogenize with water and drink a bottle cup of it in the morning in empty stomach	[40]
	*Acacia seyal*	Tundukiyac (O)	Barks	Gum from bark is chewed	[35]
	*Albizia amara*	Ondoddee (O)	Barks	Bark is chewed	[35]
	*Tamarindus indica*	Mala (B)	Fruits	Chopped, dispersed in water and the suspension is drunk	[42]
	*Cicer arietinum*	Shinbira (A)	Seeds	The dried seeds germinate, then eat them with an *Allium sativum* bulb	[26]
	*Entada abyssinica*	Ambalta (O)	Barks	The bark ground along with rhizome of *Zingiber officinale* and bulb of *Allium sativum* and chewed once a day for few months	[22]
	*Tamarindus indica*	Ged-Kinin (So)	Fruit/pulp	Infusion of the fruit/pulp kept overnight and drunk after taking goat soup	[33]
	*Senna italica*	Salamaki (So)	Leaves	Dried leaves powdered and boiled with water and drunk after adding goat or camel milk	[33]
*Flacourtiaceae* (1)	*Flacourtia indica*	Agnaneshewe (B)	Fruits	Eaten as it is	[42]
*Lamiaceae* (1)	*Ajuga integrifolia*	Anamuro (Hal)	Leaves	The fresh whole parts of plant crushed, the liquid is filtered & drunk it	[32]
*Loganiaceae* (1)	*Buddleja polystachya*	Amfar (A)	Leaves	Juice in empty stomach	[43]
			Leaves	Maceration/decoction taken orally once daily for seven days	[37]
	*Melia azedarach*	Almim (B)	Leaves	Leaves boiled with water and drunk	[42]
	*Azadirachta indica*	Neem (A)	Leaves	Fresh apical leaves (buds) are pounded and mixed with water (soaked) and the filtrate drunk. Lemon and salt and sometimes sugar are added	[33]
			Leaves	Grinding, chewing, boiling, liquid form	[44]
	*Skebergia capensis*	Lol (A)	Barks	Infusion of fresh pulverized bark	[29]
*Moraceae* (1)	*Ficus sur*	Oda’a (Had)	Fruits	Dry fruits pounded, powdered and then mixed with honey and taken orally twice	[34]
*Myrtaceae* (1)	*Syzygium guineense*	Duwancho (O)	Leaves	Well powdered leaves are taken with cold tea	[35]
*Oleaceae* (1)	*Olea europaea*	Awlie (T)	Bark	Boil it in water and drink the fluid	[30]
*Phytolaccaceae* (1)	*Phytolacca dodecandra*	Endod (A)	Roots	Fresh roots of *Phytolacca dodecandra* L’Herit. crush, squeeze then drink	[26]
*Polygonaceae* (1)	*Rumex abysinicus*	Mekimeko (A)	Roots	Dried roots of *Rumex abysinicus* boiled with butter and taken orally	[45]
*Ranunculaceae* (1)	*Clematis simensis*	Tauta (Ku)	Roots	Grinding fresh roots of *Clematis simensis* and giving a fingertip of this nasall	[40]
*Rutaceae* (1)	*Ruta chalepensis*	Xenadame (A)	Leaves	Leaves powder is mixed with water and drunk in the morning before breakfast for 3 days	[35]
*Sapindaceae* (1)	*Dodonia angustifolia*	kitkita (A)	Seeds	Grind and eat it with honey	[30]
*Solanaceae* (3)	*Capsicum frutescens*	Kariya (A)	Seeds	Eating the dry seeds mixing with foods	[32]
	*Datura stramonium*	Manjii (O)	Fruits	Powdered fruit of *Datura stramonium* is mixed with honey and three to four spoons are eaten with pounded *Allium sativum*	[22]
	*Withania somnifera*	Gzawa (A)	Roots	Dried roots grounded and boiled and drunk after adding goat/camel milk	[33]
*Urticaceae (2)*	*Droguetia iners*	Yewoba medihanit (Aari)	Leaves	Leaves chopped and mixed with *Premna oligotricha* and boiled together one glassful drank	[46]
	*Urtica simensis*	Sama (A)	Roots	The crushed the roots and dried the mixed with fresh water, drink one glass of it and drink much amount of milk	[29]
*Verbenaceae (1)*	*Lantana trifolia*	Yewoba medihanit (Aari)	Roots	Root chopped and soaked with water and mixed with local alcoholic drink (Areke)	[46]
			Roots	Maceration, taken two doses orally for one day	[37]

*A*  Amhargna, *O* Oromigna, *T* Tigerigna, *B* Bertagna, *Had* Hadigna, *So* = Somaligna, *Ku* Kunamaigna, *Hal* Halabigna

^a^Number of species studied by family in parentheses

**Fig. 1:**
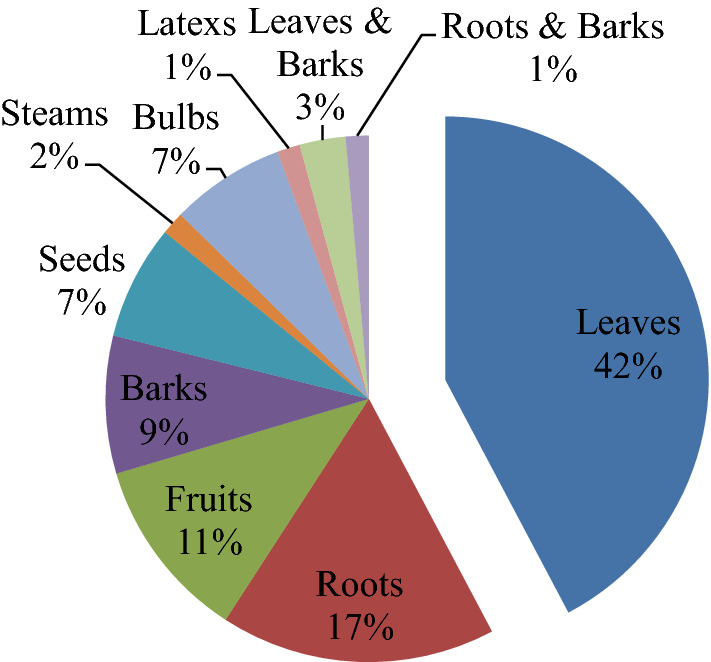
Frequency distribution of plant parts used to prepare remedies

The use of plant components and the manner in which they are prepared are limited by their availability and indigenous people's expertise [20]. According to the results of the analysis of the plant parts used, traditional healers used the leaves the most, accounting for around 42% of the total plant parts used (Fig. 1). Damage to medicinal plants caused by leaves harvesting is negligible when compared to other components [21]. The majority of these anti-malarial botanicals are employed as monotherapies, but others are utilized in combination therapies. The combination of *Entada abyssinica* barks, *Zingiber officinale* rhizomes, and *Allium sativum* bulbs chewed once a day for a few months to treat malaria is an example of a multi-herbal combination [22].

According to the review, crushing, powdering, macerating and decoction were the most common remedy formulations, and oral administration was the most common mechanism of route of delivery. In the preparation of remedies, water and various ingredients such as honey, butter, salt, coffee, tea, and milk were frequently employed. It was believed that the additions would either reduce toxicity or increase flavor. A variety of plant species used to treat malaria in Ethiopia has also been found in other African countries. Anti-malarial such as *Rumex abyssinicus* and *Vernonia amygdalina* have been utilized in Kenya [23], *Carica papaya* and *Azadirachta indica* have been used in Southwest Nigeria [24]. Many similar examples exist amongst those documented in Table 1.

### Scientific studies into the anti-malarial activity of Ethiopian medicinal plants

In vivo anti-malarial properties of extracts from 38 plant species studied in mice with rodent *Plasmodium* species were indicated for the treatment and/or prevention of malaria (Table 2). Seven studies (16%) indicated very good activity (suppression rate of 50% at 100 mg/kg body weight/day), sixteen studies (37%) reported good activity, and twenty studies (47%) found moderate activity. All in *vivo* experiments have been conducted using the 4-day suppressive test [47] and the Rane (curative) test [48]. *Aloaceae* and *Asphodelacea* were the most studied plant families. It is possible that this is an account of the *Aloaceae* family, which is found in every floristic region of the country [49]. Aerial parts, leaves, leaf latex, rhizomes, roots, stem bark, fruits, and seeds were among the plant parts examined for anti-malarial activity. The crude extract of the plants was employed in the majority of the studies (76%). *Acanthus polystachyus* [50, 51], *Aloe debrana* [52, 53], *Combretum molle* [54, 55], *Croton macrostachyus* [56, 57], *Clerodendrum Myricoides* [53, 58] and *Dodonaea angustifolia* [53, 59, 60] are examples of plant species that have been studied by more than one author. *Echinops kebericho*, *Artemisia abyssinica*, *Aloe megalacantha*, *Carica papaya*, *Combretum molle*, *Croton macrostachyus*, *Ajuga remota*, and *Dodonaea angustifolia* are some of the plant species listed in Table 2 that have anti-malarial activity, which supports the traditional uses indicated in Table 1. In experiments using a methanolic extract of the leaves, the most active species was *Ajuga remota* ('Akorarach' in Amharic; 'Etse-Libawit' in Ge'ez), which provided a high suppression of parasitaemia of 77.34% with an oral dose of 100 mg/kg [61]. Despite the fact that it is often suggested that more polar solvents such as water, methanol, and ethanol be used only in traditional preparations [62]. Surprisingly, the anti-malarial activity of most of the plant species studied matched to high polarity (methanol) plant extracts in most studies. According to Lipinski's laws of 5 [63], this is beneficial because it allows therapeutic components to absorb through the gut lumen into the circulatory system, where they are needed. As a result, active compounds work through cell surface receptors, with polar components providing clinically relevant potency in vivo.

**Table 2:** in vivo anti-malarial activity of Ethiopian medicinal plants

Family^a^	Plant species	Part (s) used	Parasitemia Inhibition with each extract and dose used for the treatment of the malaria infected mice (dose in mg/kg body weight)	Antimalarial activity	Strain of *Plasmodium* tested	Safe dose to non-infected mice (mg/kg body weight)	References
*Acanthaceae* (2)	*Adhatoda schimperiana*	Root	Hydroalcoholic crude extract, 53.6% at 600 mg/kg	Moderate	*Pb*	2000	[50]
	*Acanthus polystachyus*	Leave	80% methanol extract, 49.25% (400)	Moderate	*Pb ANKA*	2000	[51]
	*Acanthus polystachyus*	Root	80% methanol, 51.48% (400)	Moderate	*Pb ANKA*	2000	[64]
*Aloaceae* (5)	*Aloe pirottae*	Latex	80% methanol extract, 47% % at 600 mg/kg	Moderate	*Pb ANKA*	2000	[65]
	*Aloe citrina*	Leave latex	Latex extract, 60.59% (400)	Good	*Pb ANKA*	5000	[66]
	*Aloe weloensis*	leave latex	Leave latex extract, 66.84% at 400 mg/kg	Good	*Pb*	2000	[67]
	*Aloe percrassa*	Leave latex	Water extract, 73.6% (400)	Good	*Pb* ANKA	5000	[68]
	*Aloe megalacantha*	Leave	Leave latex extract, 56.4% (400 mg/kg)	Moderate	*Pb* ANKA	2000	[69]
*Anacardiaceae* (1)	*Schinus molle*	Seed	Methanol crude extract,66.91% at 400 mg/kg	Good	*Pb*	2000	[70]
*Asclepiadaceae* (1)	*Periploca linearifolia*	Stem bark	Methanol extract, 56.98% (600 mg/kg)	Moderate	*Pb* ANKA	2000	[71]
*Asphodelaceae* (4)	*Asparagus africanus*	Leave	Leave latex crude extract, 60.70% at 300 mg/kg	Good	*Pb*	1500	[72]
	*Aloe debrana*	Leave	Leave latex crude extract, 75.02% at 600 mg/kg	Good	*Pb*	5000	[52]
	*Aloe macrocarpa*	Leave	Leave exudate crude extract, 60% at 100 mg/kg, 67.8% at 200 mg/kg and 74.3% at 400 mg/kg	Very good	*Pb ANKA*	2000	[73]
	*Aloe debrana*	Leave	Methanol extract, 73.95% at 600 mg/kg	Good	*Pb ANKA*	3000	[53]
	*Kniphofia foliosa*	Rhizome	80% methanol extract, 51.39% (200) and 61.52% (400)	Good	*Pb ANKA*	2000	[74]
*Asteraceae* (3)	*Echinops kebericho*	Root	70% ethanol, 57.29% (500 mg/kg)	Moderate	*Pb ANKA*	5000	[75]
	*Vernonia adoensis*	Leave	methanol extract, 83.36% (600)	Moderate	*Pb ANKA*	3000	[76]
	*Artemisia abyssinica*	Aerial parts	80% methanol, 64.7 and 82.4% at 200 and 400 mg/kg	Good	*Pb*	NE	[77]
*Balanitaceae* (1)	*Balanites rotundifolia*	Leave	80% methanol, 67% (400)	Moderate	*Pb ANKA*	2000	[78]
*Brassicaceae* (1)	*Brassica nigra*	Seed	80% methanol extract, 50% (200) and 53.13% (400)	Good	*Pb ANKA*	NE	[79]
*Caricaceae* (1)	*Carica papaya*	Root & Fruit	Petroleum ether fraction of fruit rind extract, 61.78% (400 mg/kg)	Moderate	*Pb* ANKA	2000	[80]
*Combretaceae* (2)	*Combretum molle*	Stem bark	80% methanolic extract, 59.7% at 400 mg/kg	Moderate	*Pb*	2000	[54]
	*Terminalia brownii*	Bark	Methanol crude extract, 60.2% at 400 mg/kg	Good	*Pb* ANKA	2000	[81]
	*Combretum molle*	Seed	Methanol crude extract, 63.5% at 250 mg/kg	Moderate	*Pb* ANKA	NE	[55]
*Cucurbitaceae* (1)	*Zehenria scabra*	Leave	80% methanolic extract 62.5% (100 mg/kg), 72.85% (200 mg/kg) and 76.01% (400 mg/kg)	Very good	*Pb*	2000	[82]
*Euphorbiaceae* (1)	*Croton macrostachyus*	Leave	Methanol extract 91%, Chloroform fraction, 75.9% and methanol fraction, 64.2% at 600 mg/kg	Good	*Pb* ANKA	5000	[56]
	*Croton macrostachyus*	Fruit & Root	80% methanol fruit extract, 70% (400) and 87% (600), root extract, 75% (400) and 89% (600)	Moderate	*Pb ANKA*	2000	[57]
*Fabaceae* (2)	*Calpurnia aurea*	Leaves	hydromethanolic leave extract, 51.15% (60 mg/kg)	Very good	*Pb*	LD_50_ > 300	[83]
	*Indigofera spicata*	Root	80% methanol extract, 53.42% (600)	Moderate	*Pb ANKA*	NE	[84]
*Lamiaceae* (2)	*Clerodendrum Myricoides*	Leaves	Methanol fraction, 77.24% and Ethyl acetate fraction 65.21% at 300 mg/kg	Good	*Pb*	NE	[58]
	*Clerodendrum myricoides*	Leave	Methanol crude extract, 82.5% at 600 mg/kg	Good	*Pb ANKA*	3000	[53]
	*Ajuga remota*	Leave	methanol extract, 77.34% at 100 mg/kg	Very good	*Pb ANKA*	2000	[61]
	*Strychnos mitis*	Leave	Aqueous extract, 74.86% (400 mg/kg) and 95.5% (600 mg/kg), hydroalcoholic fraction, 81.49% (400) and 93.97% (600)	Moderate	*Pb ANKA*	2000	[85]
*Loganiaceae* (1)							
*Menispermaceae* (1)	*Stephania abyssinica*	Leave	80% methanol crude extract, 45.60%, Ethyl acetate fraction, 51.44% and Chloroform fraction 55.80% at 400 mg/kg	Moderate	*Pb ANKA*	NE	[86]
*Oleaceae* (1)	*Olea europaea*	Stem bark	Methanol extract, 52.40% at 400 mg/kg	Moderate	*Pb*	2000	[87]
*Rosaceae* (1)	*Hagenia abyssinica*	Stem bark	Hydroalcoholic (80% methanol), 65.29% at 100 mg/kg	Very good	*Pb*, ANKA	2000	[88]
*Rubiaceae* (1)	*Gardenia ternifolia*	Stem bark	Methanol crude extract, 59.25% at 600 mg/kg	Moderate	*Pb*	2000	[89]
*Rutaceae* (1)	*Fagaropsis angolensis*	Stem bark	80% methanol extract, 50.05% (200), 54.8% (400) and 59.7% (600)	Moderate	*Pb ANKA*	2000	[90]
*Sapindaceae* (1)	*Dodonaea angustifolia*	Root	n-butanol fraction of methanolic root extract, 67.51% (600)	Good	*Pb*	2000	[59]
	*Dodonea angustifolia*	Root	Methanol crude extract, 84.52% at 600 mg/kg	Good	*Pb ANKA*	3000	[53]
	*Dodonaea angustifolia*	Leave	acetate soluble portion of the 80% aqueous MeOH extract, 80.28% at 150 mg/kg	Very good	*Pb ANKA*	NE	[60]
*Solanaceae* (1)	*Capsicum frutescens Var. Minima*	Fruit	80% methanolic crude fruit extract, 72.65 at 100 mg/kg	Very good	*Pb*	2000	[91]
*Zygophyllaceae* (1)	*Balanites rotundifolia*	Leave	Methanol extract, 60.59% at 500 mg/kg	Moderate	*Pb*	5000	[92]

^a^Number of species studied by family in parentheses, *Pb* = *Plasmodium berghei*

### Toxicity of plants extract evaluated for their anti-malarial activity

In oral acute toxicity evaluation of the test extract, 36 studies studied toxicity assays out of 43 in vivo studies (Table 2), and 84% were found to be mortality or symptoms of toxicity was not observed, which could explain the plant's safe for folkloric use. In comparison to an in vitro investigation, the in vivo model was chosen because it takes into consideration any pro-drug effect and the likelihood of the immune system managing infection [53]. The leaves were the plant part that had the most toxicity reports. Toxicity tests have indicated that several plant species with various parts, such as *Combretum molle* stem barks and seeds [54, 55] and *Croton macrostachyus* leaves, fruits, and roots [56, 57], are harmless.

### Reported compounds characterized as anti-malarial and antiplasmodial in Ethiopian medicinal plants

**Fig. 2:**
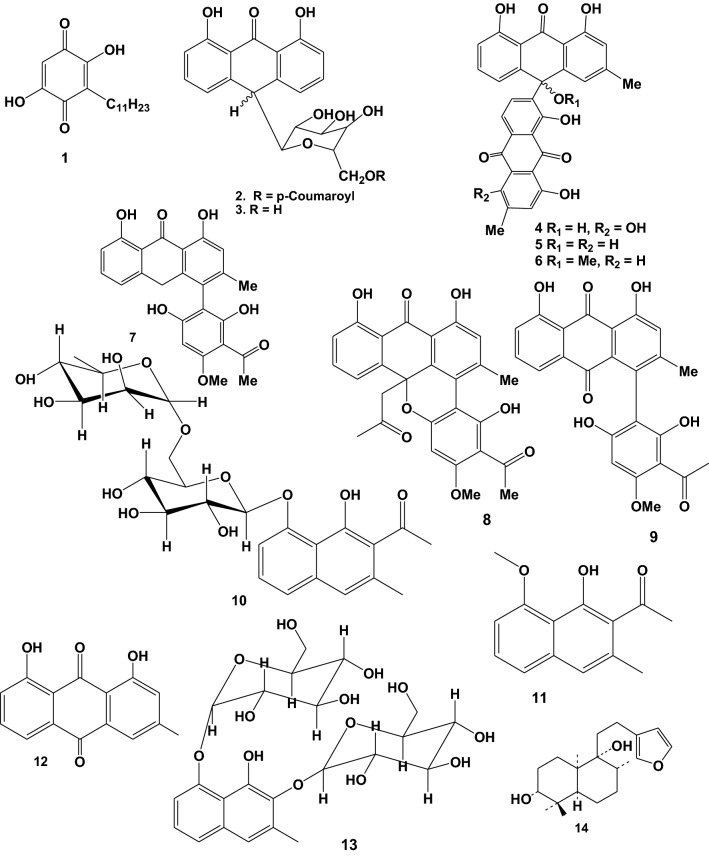

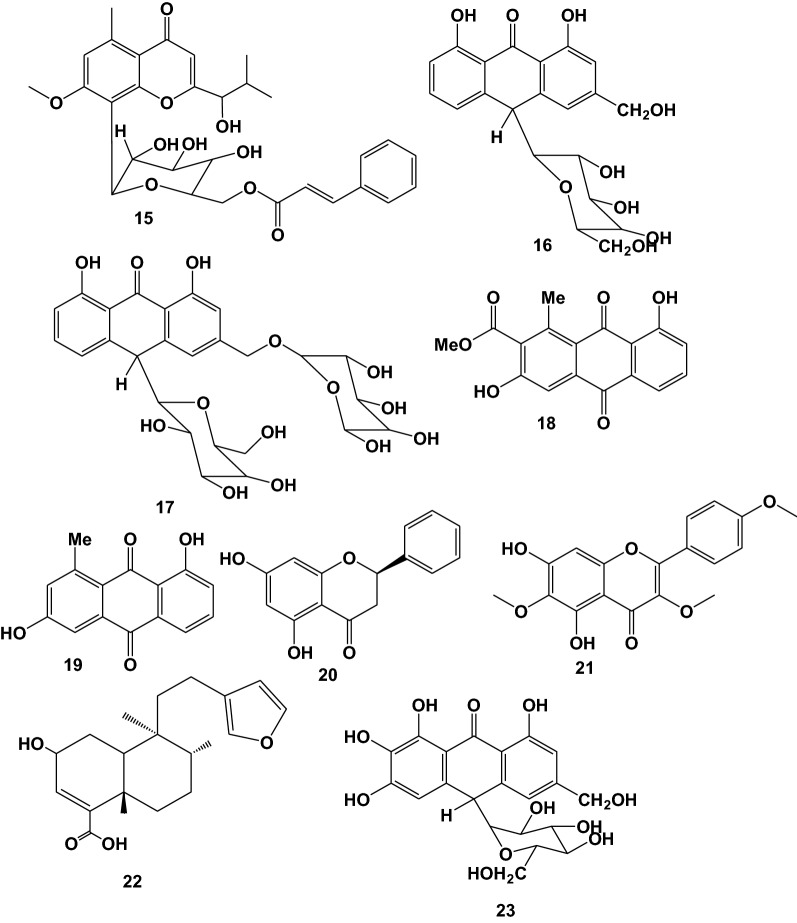
Structures of reported anti-malarial compounds from plants used in Ethiopia for malaria treatment

Ten plant species used in Ethiopian folkmedicine for malaria treatment have been shown to contain anti-malarial and antiplasmodial active compounds. The majority of the active compounds reported are anthraquinones, followed by naphthalene derivatives. Alkaloids are one of the most common types of compounds with anti-malarial activity. However, many naturally occurring nonalkaloidal compounds, such as terpenes, limonoids, chromones, xanthones, flavonoids, and anthraquinones, have anti-malarial activity when tested in various malarial models, according to a number of studies [93]. 14 (61%) of the reported compounds have been examined in *vivo*, whereas 9 (39%) have been examined in *vitro* against *P. falciparum*. There were 56% of very good, 35% of good, and 9% of moderate activity among the compounds reported. Details about these bioactive compounds are given below as well as in Table 3 and Fig. 2. The in vivo studies done by Melaku et al. [60], showed that three known compounds pinocembrin (20), flavanol santin (21) and clerodane diterpene 2*-*hydroxy-15, 16-epoxyceloda-3, 13, 14-trien-18-oic acid (22) were reported from *Dodonaea angustifolia* leaves and bio-assayed for their anti-malarial activities against *Plasmodium berghei*. According to the findings, compounds exhibited significant percent suppression of parasitaemia by 81% at 40 mg/kg, 80% at 50 mg/kg and 70% at 40 mg/kg, respectively in mice infected with *P. berghei*. Aloinoside (17) was reported from *Aloe macrocarpa* leave latex and evaluated for anti-malarial activity; it suppressed parasitemia by 100% at 400 mg/kg oral dose in *P. berghei* infected mice, and its LD_50_ was above 2000 mg/kg [73]. This suggests that this compound could be employed as an anti-malarial drug. Other phytochemicals, Aloin (16) reported in the latex of *Aloe debrana* leaves latex, inhibited infection by 78.3% at 100 mg/kg body weight and increased the survival time of mice infected with *P. berghei* [52].

**Table 3:** Anti-malarial activity of compounds reported from Ethiopian medicinal plants

Plant Species	Reported Compound (s)	Plant Part (s) used	IC_50_ or ED_50_ or Parasite suppression rate	Antiplasmodial/Anti-malarial activity	Strain of Plasmodium tested	Safe dose to non-infected mice (mg/kg body weight)	References
*Embelia schimperi*	Embelin (**1**)	Fruit	54.8% at 400 mg/kg/day	Moderate	*Pb*	2000	[96]
*Aloe percrassa*	Microdontin A/B (**2**)	Leave latex	61.4% at 400 mg/kg/day	Good	*Pb*	NE	[68]
	Aloin A/B (**3**)	Leave latex	66.8% at 400 mg/kg/day				
*Kniphofia foliosa*	Chryslandicin (**4**)	Rhizome	2.1 and 1.5 μg/ml	Very good	*Pb* D6 and W2 respectively	NE	[97]
	10-Hydroxy-10 (chrysophanol-7’-yl)chrysophanol anthrone (**5**)		1.7 and 0.7 μg/ml				
	10-Methoxy-10-(chrysophanol-7’-yl)chrysophanol anthrone (**6**)		4.1 and 1.2 μg/ml				
	Knipholone anthrone (**7**)		4.1 and 3.6 μg/ml				
	10-Acetonylknipholone cyclooxanthrone (**8**)		4.4 and 3.1 μg/ml				
	Knipholone (**9**)		55.14 and 60.2% at 100 and 200 m/kg/day	Good	*Pb*	2000	[74]
	Dianellin (**10**)		53.77 at 100 mg/kg/day				
	2-acetyl-1-hydroxy-8-methoxy-3-methylnaphthalene (**11**)	Root	15.4 μg/ml	Moderate	*Pb* 3D*7*	NE	[98]
	10-(chrysophanol-7′-yl)-10-(ξ)- hydroxychrysophanol-9-anthrone (**12**)		0.260 μg/ml	Very good			
*Aloe otallensis*	2,8-O,O-di(*β*-D-glucopyranosyl)-1,2,8-trihydroxy-3-methylnaphthalene (**13**)	Leave latex	47.29% at 100 mg/kg/day	Moderate	*Pb*	NE	[72]
*Otostegia integrifolia*	Otostegindiol (**14**)	leave	50.13, 65.58 & 73.16% at 25, 50 & 100 mg/kg/day	Very good	*Pb* ANKA	NE	[94]
*Aloe debrana*	(E)-2-(1-hydroxy-2-methylpropyl)-8-(6'-O-cinnamoyl)-β-D-glucopyranosyl-7-methoxy-5-Methylchromone (HCGMM) (**15**)	Leave latex	63.13% at 100 mg/kg/day	Very good	*Pb*	500	[52]
	Aloin (**16**)		78.31% at 100 mg/kg/day				
*Aloe macrocarpa*	Aloinoside (**17**)	Leave latex	79.1, 90.9 & 100% at 100, 200 & 400 mg/kg/day	Very good	*Pb* ANKA	2000	[73]
*Aloe pulcherrima*	Aloesaponarin I (**18**)	Root	7.8 μg/ml	Good	*Pb* D6	NE	[99]
	Aloesaponarin I (**19**)		5.0 μg/ml				
*Dodonaea angustifolia*	Pinocembrin (**20**)	Leave	77.03% and 81.00% at 20 and 40 mg/kg/day	Very good	*Pb*	NE	[60]
	Santin (**21**)	Leave	80.95% and 85.50% at 50 and				
			100 mg/kg				
	(2-hydroxy-15,16-epoxyceloda-3,13,14-trien-18-oic acid) (**22**)	Leave	60.35% and				
			70.81% at 20 and 40 mg/kg				
*Aloe pulcherrima*	7-hydroxyaloin (**23**)	Leave latex	56.2% at 200 mg/kg/day	Good	*Pb*	2000	[100]

In the studies with *Otostegia integrifolia*, very low doses of Otostegindiol (14), the active principle (25, 50, 100 mg/kg body weight), have been tested, resulting in chemosuppression of 50.13, 65.58 and 73.16%, respectively, in *P. berghei* (strain ANKA)-infected mice [94]. Because such low doses are clinically feasible for human use, efforts should be focused on the development of anti-malarial compounds with higher activity at low doses. However, because certain natural products are metabolized and the pharmacokinetics of individual natural products are frequently ignored, the likelihood that the in vitro data given (Table 3) in studies with some phytochemicals may be misleading cannot be overlooked. Compounds that are said to be active in vitro may be inactive in vivo. More pharmacokinetic studies using these phytochemicals would be tremendously beneficial, though it should be noted that most of the time, small quantities of these compounds are isolated, which limits in vivo studies. Moreover, some of the phytochemicals which have been reported to be active in *vivo*, exhibited such activities only at very high doses that may not have meaningful therapeutic use. Also, the toxicity of almost all of these purified compounds have not been be evaluated. This severely limits their potential as anti-malarial drugs in the future. Considering the importance of cytotoxicity tests, the selectivity index for all plant extracts (Table 2) and purified compounds (Table 3) has yet to be computed. The significance of the SI (CC_50_ value on cell lines/IC_50_ value against *Plasmodium* spp.) value in any study on herbal drugs and/or purified compounds is crucial for determining whether further works can be continued [95]. All these have brought limitations on some of the reported compounds being considered as lead molecules for anti-malarial drug development. Therefore, the purified compounds must be further investigated, taking into account the limitations in the development of new anti-malarial drugs and/or indicating the best anti-malarial remedies.

## Conclusion

As a result of several ethnobotanical investigations conducted in Ethiopia, a great variety of plants utilized by indigenous people to treat various ailments, including malaria, have been described. The most often used ethnobotanical plant species for the treatment of malaria were *Allium sativum*, *Croton macrostachyus*, *Carica papaya*, and *Lepidium sativum*. Leaves were used more frequently as a therapeutic preparation than other parts. The anti-malarial activity of the species investigated, as well as their potential as sources of new anti-malarial compounds and toxicities, is reviewed here. The most active species were *Ajuga remota*, *Capsicum frufescens*, *Hagenia abyssinica*, *Zehenria scabra* and *Aloe macrocapa*, which suppressed parasitaemia by 77.34%, 72.65%, 65.29%, 62.5% and 60%, respectively, at an oral dose of 100 mg/kg and an LD_50_ of above 2000 mg/kg. These are herbs that have traditionally been used to treat malaria. The compound Aloinoside (**17**) reported from *Aloe macrocarpa* leave latex and evaluated for anti-malarial activity; it suppressed parasitaemia by 100% at 400 mg/kg oral dose of *P. berghei* infected mice, and its LD_50_ was above 2000 mg/kg. This suggests that this compound could be employed as an anti-malarial drug. Malaria control efforts and resources have expanded in Ethiopia, where the burden of malaria is the highest due to the country's vast population and geographical setting. In the light of these facts, this review focuses on Ethiopian medicinal plants used to treat malaria, as well as compounds purified from them, in the hope of helping eliminate the disease. Because it is hoped that the discovery of active compounds in plants would lead to the development of more effective drugs that are both economical and accessible to rural communities at the greatest risk of disease morbidity. However, no further investigation of the efficacy of several plant species that have been described as anti-malarial could be found. More studies are needed to identify and develop successful novel drugs that could be used in broader malaria eradication efforts.

## Data Availability

Nil.
